# Frames and counter-frames giving meaning to palliative care and euthanasia in the Netherlands

**DOI:** 10.1186/s12904-021-00772-9

**Published:** 2021-06-03

**Authors:** Baldwin Van Gorp, Gert Olthuis, Anneleen Vandekeybus, Jelle van Gurp

**Affiliations:** 1 KU Leuven, Institute for Media Studies, Parkstraat 45 box 3603, 3000 Leuven, Belgium; 2 Radboud University Medical Center, Radboud Institute for Health Sciences, department IQ healthcare, PO Box 9101, 6500 HB Nijmegen, The Netherlands

**Keywords:** Framing, Social representations, Euthanasia, Palliative care, The Netherlands

## Abstract

**Background:**

Based on the case of palliative care and euthanasia in the Netherlands, this paper presents an analysis of frames and counter-frames used in the ongoing public debate about these two intertwined topics. Each (counter)frame presents a cultural theme that can act as a prism to give meaning to palliative care and/or euthanasia. Each frame comprehends a different problem definition, consequences and policy options. Typical word choices and metaphors are identified that can evoke these frames and the underlying reasoning. The frames do not belong to a specific stakeholder but a pattern can be seen in their use that is related to interests and ideology.

**Methods:**

An inductive framing analysis was conducted of 2,700 text fragments taken from various Dutch newspapers, websites of stakeholders and policy documents in the period 2016–2018. After an extensive process of thematic coding, axial coding, selective coding and peer review seven frames and seven counter-frames about palliative care and euthanasia were constructed. Fifteen experts in the field of palliative and/or end-of-life care commented on the overview during a member check.

**Results:**

Two frames about palliative care were constructed: the *Fear of death* frame, which stresses the hopeless ‘terminality’ of palliative care and the *Heavy burden* frame, in which palliative care is too big a responsibility for the relatives of the patient. In addition, two counter-frames were constructed: palliative care as a contributor to *Quality of life* and *Completion*. With regard to euthanasia, five frames were identified that lead to a problematising definition: *Thou shalt not kill*, *Slippery slope*, *Lack of willpower*, *I am not God*, and *Medical progress*. Five counter-frames offer a non-problematising definition of euthanasia in the debate: *Mercy*, *Prevention*, *Triumph of reason*, *Absolute autonomy*, and *Economic utility thinking*.

**Conclusions:**

The debate in the Netherlands on euthanasia and palliative care is characterized by a plurality of angles that goes beyond the bipolar distinction between the pros and cons of euthanasia and palliative care. Only with an overview of all potential frames in mind can an audience truly make informed decisions. The frame matrix is not only useful for policy makers to know all perspectives when joining public debate, but also to health care workers to get into meaningful conversations with their patients and families.

**Supplementary Information:**

The online version contains supplementary material available at 10.1186/s12904-021-00772-9.

## Introduction

If the suffering from serious illness becomes more and more prominent, palliative care can be initiated to relieve and alleviate the symptoms. Euthanasia is the practice whereby a medical practitioner, at the explicit request of a patient, actively ends that suffering. Such last phase of life decisions require sense making and interpretation of objective facts and a subjective sense of, amongst others, incurability, suffering, and unbearability. This demands moral consideration and ritualisation by individuals as well as cultures. In many countries, there is an ongoing social debate on the (adequate) application of palliative care and euthanasia, which in many cases already resulted in legal frameworks of some kind. The positions vary, including that euthanasia is the solution to a suffering that is problematised, or conversely, that palliative care is the solution whereas euthanasia is the central concern. In sum, it is about the “legitimacy of the competing sets of criteria by which a factual situation will or will not qualify as a genuine social problem” [1].

Palliative care and euthanasia are sensitive taboo topics. This means that at least some views on care at the end of life remain unspoken in the social debate. The applied words and images evoke particular associations and often imply a whole train of thought, with underlying values and judgments. The aims of this study are, first, to make these implicit messages explicit, second, to bring order to them, and, third, to bring more obscured perspectives to the fore. What exactly is seen as the problem may differ and also lead to different solutions. Mapping out the reasoning behind the words and images used in the various media should lead to an in-depth comprehension of the debate, for example, understanding which views are juxtaposed and which reinforce each other. This overview should also have a certain level of abstraction so that it transcends the discussion within a certain time frame. As such, this study will map out the different interpretations and moral considerations concerning palliative care and euthanasia, and it will do this by using Social Representations Theory (SRT) and Framing-Counter-framing Theory (FCT), which both play a determining role in the formation of shared meanings and the social construction of reality [2].

### Social representations and framing

Moscovici [3] described social representations as ideas and beliefs shared by members of a particular culture that help them to make something “unfamiliar familiar”. This can firstly be done by reducing these complex, disturbing or ambiguous ideas to a system of well-known categories (= *anchoring*). For example, conservative voices in the Spanish press presented palliative care on moral grounds as actions to prevent euthanasia, as long as it is not a “cover-up” of euthanasia [4]. On the basis of a Finnish interview study, Jylhänkangas et al*.* [5] referred to the “incompatibility” of euthanasia and the role of the physician. In the same regard, the discussions of physicians in the Canadian press were mainly about the place of euthanasia in mainstream medicine, whether it is possible to make a clear distinction between euthanasia as a category and other medical practices in patients’ last phase of life and palliative care advocacy [6]. Secondly, in the process of *objectification*, an abstract and possibly imprecise idea becomes a more easily accessible reality. A possibility in objectification is that an actual object is chosen in order to metaphorically reduce the complex issue to a clearer perception, such as the ‘slippery slope’. An important conclusion of this objectivation might be that no imposed measure or precautionary thoughtfulness can prevent that; once euthanasia is allowed, it will open a door to misuse it.

Goffman [7] takes up a very similar position to Moscovici and states that a *frame* formulates an answer to the question: “What is it that’s going on here?” Depending on the frame, the meaning of euthanasia and palliative care changes, in each case with a different definition, cause, consequence, solution and moral judgement. It can be expected that different positions will be expressed in the debate on euthanasia [8]. Frames bring structure to these social representations and give rise to the compilation of an exhaustive series of mutually exclusive *frame packages* [9]. A frame analysis is therefore not about identifying a number of single elements, but aims to provide a complete overview of perspectives that are common in a particular culture.

In addition, FCT proposes that frames mainly contribute to defining certain social phenomena as issues, and therefore as problems, whereas frames also can do the opposite, namely define these very same phenomena as not problematic. If a frame results in the deproblematisation of an issue, it is called a counter-frame [10, 11]. Note that deproblematising does not equal ‘not problematic’, as for particular stakeholders the deproblematisation of euthanasia will be problematic anyway. Therefore, to gain insight into a debate, it is essential to distinguish between the problematising frames of the deproblematising counter-frames.

What FCT also adds is the distinction between the logical building blocks, from causes to solutions, which are called *reasoning devices* [12]. Reasoning devices constitute the underlying argument that does *not* need to be explicitly present in communication and which indicates the power of framing: the associative human brain completes the missing parts. The identifiable elements that can activate this line of thought are labelled *framing devices*. It concerns metaphorical language, lexical choices, catch phrases and exemplary illustrations [12].

### Research question

In this study, we want to gain insight into *all* possible lenses that are used to look at palliative care and euthanasia in Dutch public discourse (for a socio-cultural history of palliative care and euthanasia in the Netherlands see Additional file 1). This study adds to the existing literature a coherent description of palliative care frames *and* euthanasia frames used in Dutch public debate, a close connection that is also present in the Dutch practice where palliative care and euthanasia are heavily intertwined (see Additional file 1). Although there are some crucial building blocks already identified in the literature (e.g., [13–15]), the logical connection between them as presented in the frame packages is not been described in the literature before.

This frame analysis of the public debate will largely be done by studying Dutch news media, given that the social debate between proponents and opponents of euthanasia involving controversial groups receives significant attention in Dutch news media [16]. This leads to the following research question:

RQ: What are the problematising frames and deproblematising counter-frames that are used to communicate about palliative care and euthanasia in the Netherlands?

## Methodology

### Sample

The researchers opted for purposive sampling, prioritizing diversity rather than representativeness of the sample of analysis material. The aim, after all, is to gain insight into the whole range of possible frames, and not into the degree of use, which would presuppose a probability sample. Several cycles of collecting analysis material took place until a point of saturation was reached for each frame, i.e., it needed to be sufficiently clear what each frame stood for, what reasoning it represented and how it could be recognized.

Collecting material started on 1 January 2016 and ran until 31 July 2018. The online press database LexisNexis (www.lexisnexis.nl) was consulted to select newspapers and magazines, applying the following search terms (in Dutch): “euthanasia”, “assisted suicide”, “completed life”, “palliative care”, and “end-of-life”, in combination with “the Netherlands”. The same key words were used in Google to find additional material to analyse. Subsequently, snowball sampling based on additionally found search terms was conducted with an eye toward mapping the breadth of the debate. In total, 467 publicly-available texts were coded: 109 newspaper articles, 25 articles in online news-only webpages, 32 magazine articles, 49 op-ed contributions and blog posts, 31 compilations of reader reactions, 6 reports on radio or television, 31 contributions to public and commercial broadcasting webpages, 97 messages posted on Twitter, 17 policy documents and 70 contributions to the internet websites of stakeholders such as NVVE (the Dutch Association for a Voluntary end of Life) and www.mantelzorgelijk.nl (an online platform for and by informal caregivers).

### An inductive frame analysis

Within framing research, various methods are commonly used (see [17]). In this case, a manual inductive method was chosen [11, 18]. The qualitative content analysis consisted of three phases: thematic coding, axial coding and selective coding. These phases were run in parallel until no additional text fragment was found that could not be placed within any of the defined frames.

First, the first and third author did *a thematic analysis* in which they systematically coded and discussed each statement or image concerning euthanasia or palliative care. A total number of 2,700 fragments selected from the 467 texts were assigned at least one code, indicating whether it gave an answer to the question of how euthanasia or palliative care can be defined, what the cause is, the consequence or the solution for that (de)problematising definition, and what moral judgment is attached to it (data and codings are available on request from the authors). Additional attention was paid to *framing devices*, i.e. the textual elements that can evoke a particular frame. For instance, a newspaper article [19] dealt with a discussion about a drug that could be used to “commit” euthanasia. On the one hand, some of the selected lexical choices indicated that the unaccompanied use of it by lay people would not be the expression of a well-thought-out choice process, but of an “ill-advised death wish” or an "impulsive suicide". On the other hand, there was an emphasis on the absolute autonomy of the individual, who should not be forced to live any longer (“coercion to live “). However, that high degree of individualisation was also associated with the “trivialisation of death”. The link with palliative care was discussed when it was stated that such a drug could “tempt” overburdened informal caregivers to use it “inappropriately”. All these text fragments were selected and coded so that they could be clustered in a subsequent phase.

Second, during a phase of axial coding, the selected fragments were grouped according to the attached codes. The intention here was to look for patterns and come up with logical arguments. For example, if euthanasia is the result of the most personal choice of the individual involved, then it could be argued that the government, caregivers and faith institutes must respect this individual’s will unconditionally. These types of reasoning were systematically composed, and further substantiated with, among other things, catchphrases and metaphorical language that contribute to making these arguments convincing.

Finally, all logical building blocks were put into a *frame matrix*, in which each row represents *a frame package*, a logical chain of reasoning and framing devices. Key criteria were that each step in the reasoning sounded logical and that the frames were exclusive. Important to this was the search for the name of the central culturally embedded frame that made each package into a whole. After all, the naming of a frame is also a form of framing on the part of the researchers. For example, one of the frames was referred to as "Thou shalt not kill called," despite the caveat that this frame also includes non-religious positions. Several preliminary versions of the frame matrix were discussed by all authors, each of which started the next cycle of analysis.

### Member checking

The third author conducted fifteen member-checks with experts from various sectors involved in palliative care and/or euthanasia: a psychiatrist; a clinical psychologist-psychotherapist; two professors in elderly care medicine; a professor in philosophy; a professor public health; three oncologists; two coordinators of a network for palliative care; a director of a patient association; a journalist/author; a post-doctoral researcher in end-of-life decision making; and a politician. These member checks ensured that most if not any researcher bias could be compensated for.

During this member check, the frames and counter-frames were presented one by one in the form of key messages based on the frame matrix. The informants were asked to evaluate them: Do you recognise these key messages in the social debate? Are they built up logically? etc. This feedback section did not take place at a final stage, but throughout the analysis process, in order to encourage interaction between the researchers and the experts.

All member checks were recorded and transcribed verbatim. The experts allowed written informed consent beforehand, and they were given the opportunity to respond to earlier drafts of this article.

## Results

The frame analysis led to the reconstruction of seven problematising frames and seven associated deproblematising counter-frames (see Table 1, for a full overview see Additional file 1). In the following section, all frames (indicated by an A) are described first. The opposing counter-frames (indicated by a B) are presented in the second part.

**Table 1:** Overview of frames that result in a problematising definition or a deproblematising definition of palliative care and euthanasia

Problematizing frame	Definition	Deproblematizing counter-frame	Definition
1A Fear of dying	Palliative care is terminal care, the beginning of the end	1B Quality of life	Palliative care is multi-faceted support for patients and relatives
2A Heavy burden	Palliative care is a difficult responsibility for the patient's relatives	2B Completion	Palliative care is a significant, valuable and enriching experience
3A Thou shalt not kill	*Committing* euthanasia is a crime of humanity against life, a criminal offence	3B Mercy	*Granting* euthanasia is an act of charity for a suffering fellow person
4A Slippery slope	Euthanasia is the light-minded liberal solution in a society	4B Prevention	Euthanasia is guiding people who want to die in a well-considered way (= donorship)
5A Lack of willpower	Euthanasia as a sign of refusing to see suffering as part of life	5B Triumph of reason	Euthanasia is a victory of human reason over death, an act of heroism
6A I am not God	Euthanasia is a heavy decision about another person's end of life	6B Absolute autonomy	Euthanasia is a decision about one's own moment of death
7A Medical progress	Euthanasia deprives man of opportunities offered by medicine	7B Economic utility thinking	Euthanasia puts an end to the untenable health care costs

### Seven problematising frames

With regard to palliative care, there are two frames that lead to a problematising definition in the debate. On the one hand, palliative care is perceived as ‘terminal’ care in the frame *Fear of death* (1A). Palliative care, “the road to the final destination” [20], generates thoughts such as “there is no more hope” and emotions such as uncertainty and loneliness. The parties involved seem to evade or delay talking about either death or palliative care. Additionally, palliative care takes a position outside regular medicine: “[X] hears from nurses that they do not conduct end-of-life conversations as part of their duties, because it would take away the hope or because ‘that is what the palliative team is for’” [21]. On the other hand, according to the frame *Heavy burden* (2A), palliative care is too big a responsibility for the relatives of the patient. Due to social pressure, they feel accountable, and they dedicate themselves to remain close to the patient. The extra effort this requires causes stress, indicated in the analysis material by words such as “emotionally a burden”. Sometimes, reference is made to the ‘liberation’ of the heavy palliative caregiving, as can be recognised in this phrasing:

When my mother was still alive, I didn’t dare to fantasise. She lost control of life and, out of love, I didn’t want to confront her with change. But now that barrier is gone, and I feel like I can start over again [22].

With regard to euthanasia, five frames were identified that lead to a problematising definition: *Thou shalt not kill* (3A), *Slippery slope* (4A), *Lack of willpower* (5A), *I am not God* (6A), and *Medical progress* (7A).

The basic idea of the frame *Thou shalt not kill* is that life, in whatever phase or capacity, is valuable. As a result, “committing” euthanasia is presented as committing a crime against life. The word use consists of variants and associations of a criminal act. By comparison with murder, this frame also makes it obvious that euthanasia should be punishable. The frame *Slippery slope* postulates that euthanasia is increasingly becoming a light-minded solution in a liberal society; whoever ‘is a little bit tired of life’ can receive euthanasia. In the analysis material, use was made of metaphors such as “supermarket euthanasia” [23] and “freely available: humane death” [24]. In *Lack of willpower*, euthanasia is seen as a sign of weakness, because someone refuses to accept or bear suffering as part of life. The frame can be recognised by formulations that indicate the choice for euthanasia as a simplicity solution. The starting point of the frame *I am not God* is that euthanasia involves a difficult moral decision about someone else's end of life that is passed on to another. This is done primarily to doctors, but indirectly also to relatives or to society, as the responsibility for the judgment and the act that requires euthanasia does not belong to human beings. Seen from this frame, also a medical doctor does not necessarily feel competent about these “very complicated dilemmas”: “We are not there to kill, we are there to cure them” [25]. A medical doctor is hesitant and does not necessarily feel competent:

It is clear that the doctor has a crucial role in euthanasia. Why did the committee choose this? ‘Because the doctor, together with the judge, is one of the few anointed in society’, says committee chairman Paul Schnabel. ‘The doctor has a separate position in society, in order to tackle a number of difficult problems’ [26].

The final frame, *Medical progress* (7A) is built around the hope that medicine offers, and ultimately the hope for an eternal life. Seen from that perspective, euthanasia ‘deprives’ people of the opportunities offered by medicine.

### Seven deproblematising counter-frames

Two counter-frames offer a non-problematising definition of palliative care in the debate: *Quality of life* (1B) and *Completion* (2B). The first, *Quality of life*, departs from the assumption that there are unspoken wishes and expectations among patients and relatives about illness, care and the end of life. Reasoning from this counter-frame, the confrontation with death is entered into, not avoided, as it is in the frame *Fear of death*. Furthermore, in palliative care, issues such as “excessive continuation of medical treatment” are made negotiable. (Early) palliative care is referred to in the data as “an added value”, and it “offers more control”. Also, one “can finish life better” and even “live longer”. Palliative care increases people’s resilience, as demonstrated by this excerpt:

From that moment on, I found that I had to stay positive. […] At first, these steps were big: seeing my children getting married, becoming a grandmother. Now they have become small steps. Going on holiday with the whole family, my eldest daughter leaving home, becoming fifty [27].

Typical of this counter-frame is that defining ‘quality’ of life is open to subjective interpretation. In practice, it could be interpreted very restrictively (e.g. drinking a glass of champagne), whereas physical, psycho-social and spiritual aspects of life, suffering and dying, possibly remain undiscussed. In the second counter-frame, *Completion*, palliative care is seen as a very meaningful and valuable period for both the (informal) caregivers, who can say goodbye in a satisfactory way, and the patients who have the opportunity to look for an acceptable end for their life stories. The idea is that palliative care usually involves “direct contact and exchange between people” [28], allowing them to deepen their relationships. Like the frame *Heavy burden*, the counter-frame *Completion* places the (informal) caregivers in a central position. In the counter-frame, however, they are also grateful for the significant role they can play. Palliative care contributes to their self-development. In the data, palliative care is referred to as “a pure gift”.

Five counter-frames are identified to deproblematise euthanasia: *Mercy* (3B), *Prevention* (4B), *Triumph of reason* (5B), *Absolute autonomy* (6B), and *Economic utility thinking* (7B). The counter-frame *Mercy* is based on the assumption that life is valuable, but if it becomes an unbearable agony without the prospect of significant improvement, it is a moral duty to release people from their suffering at their request. From the underlying moral foundation mercifulness, also defined by lexical choices as “compassion” and “caring”, the emphasis lies on the responsibility of society to intervene when people can hardly bear their suffering. The analysis material often emphasises the ‘goodness’ of euthanasia, for example: “He drinks it laughing, as if it is Pernod [aniseed]” [29]. In the counter-frame *Prevention,* both palliative care and euthanasia function as an avoidance of something that is considered less desirable: unnecessary suffering, deterioration, old age, meaninglessness of life, and suicide. As such, euthanasia is perceived as a well-considered and responsible way to guide people with “a death wish”: “Better the End-of-Life Clinic than that people throw themselves in front of the train” [30]. Policy must follow this societal trend by regulating and facilitating euthanasia. This perspective is expressed in the next quote:

It is part of the Dutch identity. We do not want everyone to commit suicide. But we want people, who themselves think that their end of life has come, to be able to make good use of opportunities to die [31].

Whereas the frame *Lack of willpower* defines euthanasia as a sign of weakness, the counter-frame *Triumph of reason* presents the intervention as a sign of strength, as the data set refers to euthanasia as a “courageous” choice. Those directly involved, their environment and the facilitating society all gain a victory over death as they can plan and organise the moment of death. At the frame’s base lies the prospect of a deterioration process. This can imply an agony from which one wants to protect not only herself or himself, but also the relatives.

In the counter-frame *Absolute autonomy*, euthanasia is a decision about one's own moment of death, which must be respected and granted by others, regardless of a person’s situation. In contrast to *Triumph of reason*, in which caution and regulation from the outside are central reasoning devices, in this frame it is the directly involved person who decides. The right to choose the time of death belongs only to the individual. In the data set, this is expressed through terms such as “taking ownership”. Based on the moral value of self-determination, individuals can lead their own life at their own discretion. This should be distinguished from the idea of ​​ ‘relational autonomy’ because, in this frame, everyone can determine their own end, without having to consider the impact on others. According to this counter-frame, different routes are possible, such as “assisted suicide” in unbearable and hopeless suffering, and the termination of a “completed life” with the help of an expert, also known as a “death counsellor”. Furthermore, there is the “autonomous route” without guidance, for example via “powder euthanasia” or a “last-will pill”.

A fifth and last counter-frame that deproblematised euthanasia was less prominent in the data, namely *Economic utility thinking* (7B). This counter-frame is based on a rational cost–benefit analysis, so that in our “performance-oriented culture” [32] something is of use only if the yield is greater than the cost involved. Investing in the ever-increasing group of ‘unusable’ people is costly and unprofitable. Granting euthanasia in a flexible way would be a solution in that respect.

### The relationship between the framing of euthanasia and of palliative care

The framing of euthanasia on the one hand and palliative care on the other are related, although the relationship is not always straightforward. With regard to the frames that are connected with palliative care, two patterns can be distinguished. The frame *Heavy burden* does comprise a more logical step towards euthanasia, whereas the opposite is true for *Fear of dying*. The counter-frame *Quality of life* primarily means that palliative care guarantees more quality at the final stage of life, and as such euthanasia might not be a logical step to take. Also with *Completion*, there is a deproblematisation of palliative care, which makes euthanasia a less obvious choice, although an argument in the opposite direction is also conceivable.

When it comes to the frames that give meaning to euthanasia, three patterns can be distinguished. First, there are the frames that result in a problematising definition of euthanasia. The fact that good palliative care can offer an alternative applies to all of them. For instance, in the frame *Thou shalt not kill*, society must focus on good palliative care to prevent people from suffering and longing for death; in the frame *Lack of willpower*, palliative care providers can make human suffering more bearable; and in the frame *I am not God* it offers help by being involved in the decision making of euthanasia.

Second, with regard to the identified counter-frames, euthanasia is not a problem and, as such, it can be inherently part of palliative care. For example, within *Mercy*, euthanasia can be included in palliative care, as the first is defined as the humane way of ending suffering at the request of the person by “providing” euthanasia. Alternatively, palliative care can opt for ‘palliative sedation’, which means that there is no interference in the natural course of dying. “Unnecessary” suffering is “softened” and “made tolerable”, through the attention, warmth and proximity that palliative care offers. According to the counter-frame *Prevention,* policy must follow this societal trend by regulating and facilitating euthanasia. On the other hand, palliative care can be seen as a prevention of euthanasia. Palliative care offers the opportunity for seriously ill people to end their lives in a dignified way. Euthanasia may be labelled as a merciful death, but it also involves intervention in the natural course of the end of the life process. The reasoning in *Triumph of reason* is that euthanasia can allow one to die in a dignified way, offering peace and strength to sustain life, possibly even longer. Palliative care providers can supply support through the proactive organisation, preparation and management of death.

A third possibility is that euthanasia is not problematised, as is the case with *Economic utility thinking*, and that the emphasis on palliative care is used as a counter-frame, in order to contradict it. In extremis, however, euthanasia can be seen as a way out for the (palliative) healthcare costs. This idea can be found in statements such as “‘Voluntary’ euthanasia is a ‘tidy–up-neatly’ action by the cabinet” [26]. This deproblematising perspective can be perceived as highly problematic at the moral level because it places older and sick people in a rational economical context, which suggests that they are useless and costly. As a result, they can feel pressure to request euthanasia. The framing of palliative care in terms of *Quality of life* or *Completion* can offer full alternatives, in such a way that they act as counter-frames in the debate. Palliative care, combined with pain-relief medications, can help to overcome the choice dilemmas as included in problematising framing.

### Combination of frames and counter-frames

In order that a text would contain only one frame, none of the identified frames or counter-frames were applied in isolation. For instance, *Thou shalt not kill*, *Slippery slope*, *Lack of willpower*, and *I am not God* are used in combination. As such, they confirm and reinforce each other. In the debate in the Netherlands, they are also combined with arguments involving a denial of the deproblematising counter-frames. The frame *Slippery slope*, for example, appears in texts in which societal challenges, such as aging, savings and the impoverishment of care are addressed, and *Economic utility thinking* is questioned. In this example, reference is made to the *Slippery slope* and *Economic utility thinking* raises objections:

We have placed strict restrictions on the provision of medicines in our country because we find them scary. Would we then let go of that when deciding on life and death? […] I share the fear of the elderly that eventually euthanasia can be forced upon them if they ‘no longer matter’ [33].

Furthermore, the counter-frames *Absolute autonomy* and *Triumph of reason* are applied together as euthanasia is presented as one's free choice to die in dignity with complete personal control. Finally, the frame *Medical progress* and the frame *Fear of death* manifest jointly, as palliative care is highlighted as the termination of life and the deprivation of hope, making it a topic to be postponed or avoided as long as possible.

### Frame ownership

None of the described frames belong exclusively to a particular actor. However, there seems to be a pattern in the analysed texts, in that sense that opponents of euthanasia, mainly the Christian Dutch Physicians’ Alliance (NAV), and the Dutch political party Christen Union (CU), most commonly used the problematising frames, while proponents, such as the Dutch political party Democrats 66 (D66), the Cooperative Last Will (CLW), mainly cited the alternative counter-frames. On the side of the opponents, the frame *Thou shalt not kill* is strongly, but not exclusively, expressed from a religious angle and from pro-life organisations such as Scream for Life (Schreeuw om Leven). The frame *Slippery slope* was explicitly used by some actors, for example by psychiatrists, who were concerned about the expansion of euthanasia to specific groups of vulnerable people, such as people with dementia. On the other hand, in communications coming from organisations that advocate for euthanasia, such as the Dutch Association for a Voluntary End of Life (NVVE), the counter-frames *Absolute autonomy* and *Mercy* were especially present.

The use of counter-frames was not reserved for stakeholder organisations, given that they were also noticeable in the accounts of persons requesting euthanasia and their families, although in these cases *Triumph of reason* and *Prevention* seemed to be more present. Finally, *Quality of life* was prominent in communication coming from stakeholders in palliative care. These stakeholders emphasised the attention that palliative care gives to the medical, psycho-social and spiritual changes of patients and their relatives, aimed at improving the quality of life, although there is a chance that ‘quality’ is interpreted too restrictively (see also [34]).

## Discussion

Based on the case of end-of-life care in the Netherlands, this paper presents a qualitative method with which the cultural-historical diversity of important health themes can be investigated in a meaningful way. The approach offers the opportunity to examine culturally embedded frames and counter-frames. On the basis of our analysis of Dutch public debate, we have developed an extensive matrix (Additional file 1) presenting frame packages that define thought patterns on euthanasia as well as palliative care in Dutch society. We have also presented some observations with regard to the relationship between the framing of euthanasia and palliative care, the combination of frames and counter-frames, and the ownership of particular frames. In this section we will discuss and further interpret our results in four steps. First we will relate our results to a broader outlook on the moral particularities of the Netherlands as a value pluralistic society. Then we will discuss our results within the context of other analyses of Dutch news media reports on care in the last phase of life. A third step involves the comparison of our results with analyses of news media of other countries. In a final step we will make some theoretical considerations with regard to our methodological approach.

### Value pluralism in the Netherlands

In the European Values Study [35] the Netherlands is characterized as a country that is strongly individualized, which implies a relative tolerance with regard to other people’s life choices. In the Netherlands there is a liberal and tolerant attitude in the family domain, and the sense of family duty is less marked. This means that family life is less bound by all sorts of duties and obligations towards other family members and society and the presence of a permissive approach to issues like acceptance of one’s own body and sexuality, homosexuality, and euthanasia. Furthermore, according to the European Values Study [35] this longing to be autonomous and to live one’s own experiences goes hand in hand with humanistic and altruistic values. The Netherlands belongs to ‘a participative Europe’, a group of countries that emphasize autonomy of the subject in making choices, and have confidence in others and in institutions, participating in associational life and adhering to values of authority and respecting public norms. This characterization corresponds to a comparison between US and the Netherlands on values and end-of-life care [36]. Common between these countries is their favor of individual autonomy, contrasting, however, is the Dutch manifestation of tolerance, solidarity, and pragmatism in reaction to threats to health.

Our matrix with frames (Additional file 1) accommodates a variety of values, relating to autonomy, responsibility, gratitude, humanism, non-maleficence, and well-being. The matrix shows how public discourse aligns itself with social debate and social change [37]. It seems safe to conclude that our results acknowledge the Netherlands as a society in which a plurality of values can be present. In current law, however, certain values (and thereby certain frames) are more represented than others : a focus on quality of life (frame 1B) should lead to early conversations about the end of life (e.g., [38]) in order to work towards completion (frame 2B). Palliative care is available to provide relief of suffering, but it is acknowledged that some suffering cannot be relieved. In these ‘back against the wall’-cases, euthanasia might be the only available and merciful option if supported by the person’s wish (frame 3B, frame 6B). Although the Dutch euthanasia law is primarily on the abovementioned frames, moral value pluralism implies that the variety of values that manifest themselves cannot easily be reduced to one fundamental value and there is a plurality of ways of being good [39], also concerning palliative care and euthanasia. A relevant attribute is that such pluralism allows for the complexity and conflict that is part of our moral experience. On the one hand the Dutch context of value pluralism clarifies the broad set of frame packages we found in our data, some problematizing palliative care or euthanasia, others deproblematizing them. On the other hand this value pluralistic outlook explains the fact that there is an ongoing societal debate on these complex end-of-life issues in the Netherlands since the 1960’s, between sometimes strongly opposing ethical views which are not easily brought to a consensus [16].

### Dutch news media analyses on care in the last phase of life

Van den Berg, Eliel, and Meijman [15] previously carried out an analysis of how the Dutch press portrayed palliative care and terminal care at home. They found that between 2000 and 2009 a contextual approach, with attention to politics and religion among others, gave way to a consumer-oriented perspective. From this it follows, for example, that attention is paid to patient organizations and to the importance of prevention and individual responsibility. However, their approach did not clarify the possible positions in the debate, their scope and the corresponding social representations. The work of Rietjens and colleagues [16], which focuses solely on euthanasia, offers an interesting overview of arguments pro and contra euthanasia that shows plenty of similarities with the arguments and values provided in the frames on euthanasia in this study. This study’s frames, however, contain more: they bring values and arguments in connection with possible lines of action and a particular vocabulary and metaphorical language. It also shows how current Dutch practice, in which palliative care and euthanasia are considered part of the same continuum, is built on a mix of more or less compatible frames (e.g., quality of life, completion, absolute autonomy) and disassociates from other frames (e.g., lack of willpower, medical progress). The latter, however, do not vanish in the public debate as our results underline. Two additional points are worth mentioning: Rietjens and colleagues [16] not only see media refer to euthanasia in line with the law on euthanasia, but also beyond the law. The same can be observed in the frames on absolute autonomy (in which everybody can decide to whether and when their lives should finish) and economic utility thinking (where euthanasia is considered a quick fix for rising health care costs). The most intense public debate is conducted around and beyond the limits of the euthanasia law, such as with regard to the issue of ‘completed life’ [40] and euthanasia in case of dementia [41]. Second, the frames on euthanasia might offer a start for thinking about whether euthanasia contributes to a good death. The mercy frame is probably the most outspoken frame about the goodness of death, with death being at least a better alternative than the meaningless suffering that precedes it.

### An international comparison of news media analyses

If we have a closer look at analyses of news media articles that concern euthanasia or – more broadly – medical involvement at the end of life, the most notable observation seems to be the limited nuance in the debates in those countries. The debates in countries like the United States, Australia, and the United Kingdom seem much more black and white, concerning stronger and more activist positions. A broad and morally pluralistic outlook as our Dutch frame packages (Table 1) provide seems uncommon. Perhaps not a coincidence, with euthanasia laws being recently accepted or taking effect, but two analyses from New Zealand and Canada, show resemblance with our results [42]. The study from New Zealand concerns a discourse analysis of social media in which citizens participate in the voluntary euthanasia debate. Like our results, this analysis comprehends a broad array of deeply held sociocultural values and positions that are manifest in societal debate. The debate on social media in New Zealand encompasses a complex range of positions, far more than ‘for’ and ‘against’. Nevertheless, how these values relate to the reasoning behind certain opinions on the topic is missing from the analysis. An integrated approach should examine the logical connection between the archetypes and certain socio-cultural values and reasoning devices. In Canada, the focus was on the discourses used by physicians in media reports [6]. Canadian physicians contributed to a balanced representation of palliative care and euthanasia, with pros and cons about legitimizing the euthanasia practice, with attention for the ethical differences between palliative care and euthanasia, and with some strong advocacy for high-quality palliative care in order to reduce the need for a hastened death to an absolute minimum.

A news media analysis on the press coverage of three cases of family assisted suicide in the UK [14], however, reveals a consistently supportive stance towards the issue. This outlook on family assisted suicide is produced by depictions of dying persons and perpetrators as autonomous and conscientious individuals; by idyllic portrayals of family relations; and by praising judges for their lenient verdicts. The authors detect a bias in the press coverage, which seems to present narratives of family assisted suicide in a frame of pro-euthanasia in conformity with neo-liberal ideal of self-determination (see p. 2162).

McInerney [13] identified the way in which the press represented actors in the requested death movement in Australia. The movement was predominantly represented in a heroic discourse, opponents became villains, and the terminally ill patients were portrayed as heroic victims. Recently, Lauffer, Baker, and Seely [43] analysed how the American press first portrayed 29-year-old Brittany Maynard as a tragic person when she was diagnosed with a brain tumor. Subsequently, the frame 'peaceful death' showed up in the stories, which encompassed her death wish. Finally, there was the frame 'legacy of choice', which refers to the right-to-die movement. These frames, however, goes merely beyond this concrete case-study. In contrast to our findings that comprise a much broader and more precise analysis of palliative care and euthanasia, the analysis from media coverage in the UK, Australia and the United States bring forward the strong rhetoric aspects of news media. It is difficult to draw conclusions other than careful hypotheses from these differences. Is this rhetoric, for example, due to the neo-liberal ideal of self-determination as Banerjee et al. [14] suggested?

### Some theoretical considerations

On a theoretical level, this paper demonstrates how social representations, in particular metaphors, values and ideas, can be used to attach fundamentally different meanings to important social and existential themes, in this case euthanasia and palliative care. Specific language (i.e. framing devices) can activate at the cognitive level complex, underlying reasoning devices that together form a logical whole, displayed in frame packages. Through the proposed systematic approach, it can be argued that the frame matrix is mutually exclusive and exhaustive and can form the basis for further research. For instance, performing a deductive study could reveal the extent to which the frames and counter-frames are context or culture specific, with samples coming from different countries. A cross-cultural comparative approach also makes it possible to examine how the use of problematising frames has evolved over time, for example to determine how the deproblematising counter-frames in particular ultimately led to a general acceptance of euthanasia in the Netherlands and not in other countries. This implies that it must be taken into account that different decision-making cultures can come to dissimilar conclusions, basing themselves on different framings, knowing that for the issues discussed here only a limited number of knowledge claims are available.

A further suggestion for future research would be to do a framing effects study, starting from the ecologically valid frame matrix as presented here. The way in which citizens deal with a topic cannot be deduced simply from the contents of a text. After all, a frame is only a suggestion to interpret a text in a certain way. Textual elements were identified that may trigger a specific line of reasoning at a cognitive level which, although it may be objectionable at a moral level, may still come across as a rational argumentation.

## Conclusion

Even though the regulation and attitude among the population in the Netherlands are pro-euthanasia, this does not mean that there is no debate, or that euthanasia is not problematised in any way. The debate in the Netherlands on euthanasia and palliative care is not carried out unilaterally. Clearly, there is a plurality of angles that goes beyond the bipolar distinction between the pros and cons of euthanasia. Possibly, some frames and counter-frames deserve more attention in the public debate, not so much to change the most prevalent ones, but to expand the public’s view. Only with a broad perspective can an audience make informed decisions. The same applies to palliative care. Although this topic evokes less controversy, it is a topic that is surrounded by taboos (death, suffering). Therefore, an enriched debate, with both frames and counter-frames, can also be useful to better understand the debate, to grasp its essence and to stimulate public opinion.

The practical implications of the research mainly stem from the overview of different perspectives that are prevalent in the Dutch discussion on end-of-life care, including palliative care and euthanasia. Being useful for policy makers to know all these perspectives when joining public debate, such an overview might also help health care workers to get into meaningful conversations with their patients and families who may represent one or several perspectives. It appears valuable to health care workers to know what is actually the problem, and that they are also aware of potential deproblematising frames.

## Supplementary Information

**Additional file 1. **

## Data Availability

The dataset, i.e. the codings of the texts analysed during the current study, is available from the corresponding author on reasonable request.
