# Supplementary material for: Frames and counter-frames giving meaning to palliative care and euthanasia in the Netherlands
Source: BMC Palliat Care. 2021 Jun 3;20:79. doi: 10.1186/s12904-021-00772-9 (PMC8176618; doi:10.1186/s12904-021-00772-9)
Supplement: Supplementary file 1 — Additional file 1. [file 12904_2021_772_MOESM1_ESM.docx]

**Frames and counter-frames giving meaning to palliative care and euthanasia in the Netherlands**

Baldwin Van Gorp^1^ (*)

Gert Olthuis^2^

Anneleen Vandekeybus^1^

Jelle van Gurp^2^

^1^Institute for Media Studies

KU Leuven

Parkstraat 45 box 3603

3000 Leuven

Belgium

^2^Radboud University Medical Center

PO Box 9101

6500 HB Nijmegen

The Netherlands

(*) correspondence to [baldwin.vangorp@kuleuven.be](mailto:baldwin.vangorp@kuleuven.be)

**Annex 1:** **A concise socio-cultural history of palliative care and euthanasia in the Netherlands**

The Dutch euthanasia practice [[1](#Gordijn), [2](#TenHave), [3](#Weyers)] gradually arose from a fading taboo of death and dying, as well as a critical stance towards medicine’s technological power during the 1960s in Dutch society. An increased openness to dying fitted a Dutch society which, at the time, was secularising rapidly and which, in general, had strong feelings regarding the value of candour [[3](#Weyers)]. It was the book of JH van den Berg, ‘Medical Power and Medical Ethics’ (1969, translated in 1978), that expressed a growing discomfort with physicians unsparingly applying medical technology to continue their patients’ lives [[1](#Gordijn)]. A patient’s life was still seen as valuable, but the medical interventions were subjected to the criterion of medical futility. Patients should obtain a bigger role in decision making, which is more concerned with their quality of life. Not only discussions about refraining from action arose, but also about the double effect that normal medical care can have when pain relief has the unintended side-effect of shortening life [[4](#GordijnJanssens2000)].

The 1970s produced a sample of exemplary law cases in which termination of life on request was discussed in court. The then committee of Procurators-General aimed to test the boundaries of the law and build a national prosecutorial policy [[3](#Weyers)]. At the same time, civil society joined the Dutch Society to support a Voluntary End-of-Life initiative. From these societal discussions emerged the conflict of duties (the *Force Majeure*) that was experienced by physicians: they have the duty to relieve the patient’s intolerable suffering, but, at the same time, must protect their lives [[1](#Gordijn), [5](#vanWijngaarden)]. The Dutch Medical Association, at that time, stated that physicians could be trusted to work carefully in cases of intolerable suffering and produced a professional standard on terminating life on request [[3](#Weyers)]. All this, along with solid research on the practice of terminating life on request formed the basis of a gradual transformation from a pragmatic tolerance of euthanasia into a legally codified practice [[1](#Gordijn)]. The latter was finally issued in 2002.

From 1975, palliative care slowly gained ground in the Netherlands, with noticeable initiatives in nursing homes and, from the 1990s, in specialised hospices [[6](#GordijnJanssens2004), [7](#Janssens)]. In contrast to other national networks, the Dutch network palliative care for terminal patients included euthanasia in the practice of palliative care [[4](#GordijnJanssens2000)]. Euthanasia has always been considered as a potentially worthy end whilst receiving palliative care, but high-standard palliative care has equally been considered a necessary precondition for a euthanasia practice that was not caused by insufficient and/or inadequate care [[1](#Gordijn)]. With substantial financial impulses from the government in 1996 and 2014, Dutch palliative care rapidly professionalised.

Almost two decades after legislation, and with a professionalised euthanasia and palliative care practice at hand, the Dutch society is still confronted with a few heavily debated issues. With the euthanasia practice originally being built on beneficence and compassion of physicians, there is a slow but steady shift towards autonomy and self-determination being the most important values [[8](#vanWijngaarden)]. This means that citizens increasingly claim the right to decide where and when they want to die. This resulted in a civil ‘Death with Dignity’ movement (the association “By Free Will”) which advocates for physician-assisted death for people over 70 years old who do not have a disease, but feel that ‘life is completed and no longer worth living’ [[8](#vanWijngaarden2015)]. As these elderly do not have a disease this issue lies beyond the euthanasia law, but the societal discussion however is closely related. What lies within the boundaries of the law is the discussion on the issue of (in)voluntariness, advanced directives, and euthanasia in dementia care [[9](#deBeaufort), [10](#Miller)], and the continuing discussion on the notion of ‘unbearable suffering’ that is central to the law [[11](#Dees)].

**References**

1. Gordijn B, Visser A. Issues in Dutch palliative care: Readjusting a distorted image. Patient Educ Couns. 2000;41(1):1-5. doi:10.1016/s0738-3991(00)00123-3
2. Ten Have J, Welie J. Death and medical power: an ethical analysis of Dutch euthanasia practice. Maidenhead: Open University Press; 2005.
3. Weyers H. Explaining the emergence of euthanasia law in the Netherlands: How the sociology of law can help the sociology of bioethics. Sociol Health Ill. 2006;28(6):802-816. doi:10.1111/j.1467-9566.2006.00543.x
4. Gordijn B, Janssens R. The prevention of euthanasia through palliative care: New developments in The Netherlands. Patient Educ Couns. 2000;41(1):35-46. doi:10.1016/s0738-3991(00)00113-0
5. van Wijngaarden E, Klink A, Leget C, The AM. Assisted dying for healthy older people: A step too far? BMJ. 2017;357:j2298. doi:10.1136/bmj.j2298
6. Gordijn B, Janssens R. Euthanasia and palliative care in The Netherlands: An analysis of the latest developments. Health Care Anal. 2004;12(3):195-207. doi:10.1023/B:HCAN.0000044926.05523.ef
7. Janssens RJ, ten Have HA. The concept of palliative care in The Netherlands. Palliative Med. 2001;15(6):481-486. doi:10.1191/026921601682553969
8. van Wijngaarden E, Leget C, Goossensen A. Ready to give up on life: The lived experience of elderly people who feel life is completed and no longer worth living. Soc Sci Med. 2015;138:257-264. doi:10.1016/j.socscimed.2015.05.015
9. de Beaufort ID, van de Vathorst S. Dementia and assisted suicide and euthanasia. J Neurol. 2016;263(7):1463-1467. doi:10.1007/s00415-016-8095-2
10. Miller DG, Dresser R, Kim SYH. Advance euthanasia directives: a controversial case and its ethical implications. J Med Ethics. 2019;45(2):84-89. doi:10.1136/medethics-2017-104644
11. Dees MK, Vernooij-Dassen MJ, Dekkers WJ, Vissers KC, van Weel C. 'Unbearable suffering': A qualitative study on the perspectives of patients who request assistance in dying. J Med Ethics. 2011;37(12):727-734. doi:10.1136/jme.2011.045492

**Annex 2:** **Frame packages that define euthanasia and palliative care in the Netherlands**

| 1. Social representation | Cultural theme | Definition of the issue | Starting point of definition | Consequences | Moral values involved | Possible solutions/actions | Metaphors, choice of vocabulary |
| --- | --- | --- | --- | --- | --- | --- | --- |
| 1A Fear of dying | Fear of death and everything that makes someone think about it | Palliative care is terminal care, the beginning of the end | Palliative care confronts people with the finitude of life | There is no more hope, given up by others | Everybody dies | Pushing death further away; palliative care is no part of medicine | Death as a taboo; scary; fear of the end; |
| 1B Quality of life | Standards of comfort, health and happiness in someone’s life | Palliative care is multi-faceted support (physical, psychological, social, spiritual) for patients and relatives | (Unspoken) ideas, wishes and expectations among patients and relatives | Providing appropriate care; chance of focus only on pleasant experiences | Empowerment; support; benefit from | Timely speaking about and employing of palliative care | Needs are leading; a joint decision; drinking a final glass of champagne |
| 2A Heavy burden | A burdensome task or duty | Palliative care is a difficult responsibility for the patient's relatives | Pressure on the relatives to stay and care, which requires extra effort and turns them into tragic heroes | Relatives experience a lack of recognition; patient feels guilty | Responsibility; devotion; justice | Efforts for providing care and support adapted to the patient and the family | Difficult period; overload; feelings of guilt; recognising needs |
| 2B Completion | (Self-)fulfilment as a life purpose | Palliative care is a significant, valuable and enriching experience | The wish and urge to take a meaningful role towards relatives | Creating moments of being together; deepening the mutual band | Gratitude; reconciliation; acceptance; virtuous care | Saying goodbye in a satisfactory way; live in the here-and-now; closing the loop | The most beautiful care; intimacy; enriching; satisfaction |
| 3A Thou shalt not kill | Do not kill any man as one of the universal commandments | Committing euthanasia is a crime of humanity against life, a criminal offence | Indifference and insufficient care in society | Older and sick people become easy victims of euthanasia | Every state of life is better than non-life; life is sacred | Make euthanasia punishable | Sanctity of human life; ‘committing’ euthanasia |
| 3B Mercy | Granting grace | Granting euthanasia is an act of charity for a suffering fellow person | If life is an agony, intervention becomes necessary | Death as a rescue aid to free a person from suffering (= heroism) | Mercy; humanity; grace | Society is gracious by alleviating meaningless suffering | Compassion; the beautiful farewell; liberation; lovingly |
| 4A Slippery slope | A certain action will cause a series of consecutive events | Euthanasia is the light-minded liberal solution in a society | Society grants everything that the impulsive individual wants (= societal guilt) | The offer of euthanasia creates a demand | Libertarianism; consumer society; supply and demand | Euthanasia practice must be limited by very strict and clear regulations | ‘Euthanasiasm’; euthanasia marketing |
| 4B Prevention | Reducing the incidence of suicide | Euthanasia is guiding people who want to die in a well-considered way (= donorship) | Unregulated suicides at a late stage of live | Make sure that dying is possible in a humane and well-considered way | Inevitability; prevent worse | Policy that follows the practice; in consultation | Social urge; no Wild West; a good farewell |
| 5A Lack of willpower | A lack of perseverance typifies a weak person | Euthanasia as a sign of refusing to see suffering as part of life | Not facing suffering and pain are part of life (= victim blaming) | Euthanasia as a convenient solution to suffering | Suffering is inherent to life; accepting one's own destiny | People must show willpower until the bitter end | Enduring the pain; suffering as a part of life |
| 5B Triumph of reason | The unlimited possibilities of the human mind | Euthanasia is a victory of human reason over death, an act of heroism | Fear of and insecurity about a deterioration process | To die worthy by controlling the time and the manner | Will power; self-control; self-respect; self-efficacy | The possibility to step out of life at any moment gives rest | A courageous decision; a victory over death |
| 6A I am not God | Man feels small and insignificant, and anything but mighty | Euthanasia is a heavy decision about another person's end of life | A request for euthanasia requires a judgment and an act of a physician | The heavy task causes inconvenience, self-reproach and doubt (= victimhood) | The unwillingness to bear the responsibility for someone’s death | Doctor passes decisions on to others; clear directions and procedures | Dilemma; pressure and struggle; a ‘buddy’ |
| 6B Absolute autonomy | Individuals are able to make their own choices | Euthanasia is a decision about one's own moment of death | Life is someone's own possession (= personhood) | Someone can freely choose to step out of life, or preferably appeal to others | Self-determination; libertarianism | Euthanasia and assisted suicide via the law; respect someone's rights | Free will; the right of euthanasia; self-chosen death |
| 7A Medical progress | The belief (conviction) that medical science takes humankind further | Euthanasia deprives man of opportunities offered by medicine | Increasing number of therapeutic options to prolong life (= scientific heroism | Medical treatment ends only when natural death occurs | Belief in progress and in the possibilities of human ability | Keeping faith in in the medical ability to extend life | Trained to heal; keep alive; treatments, medications |
| 7B Economic Utility thinking | Something is useful if its return is greater than its costs | Euthanasia puts an end to the untenable health care costs | Old and sick people do not comply to societal norms (= victimhood) | An unaffordable health care system; people who experience themselves as useless | Rational cost-benefit analysis; social health standards | Euthanasia can offer a way out for healthcare costs | Materialistic efficiency thinking; idealising youth and health |
